# Circulating ACTH and Cortisol Investigations in Standardbred Racehorses Under Training and Racing Sessions

**DOI:** 10.3390/vetsci12050493

**Published:** 2025-05-19

**Authors:** Cristina Cravana, Pietro Medica, Esterina Fazio, Katiuska Satué, Giacoma Brancato, Deborah La Fauci, Giuseppe Bruschetta

**Affiliations:** 1 Unit of Veterinary Physiology, Department of Veterinary Sciences, Messina University, Polo Universitario Annunziata, 98168 Messina, Italy; ccravana@unime.it (C.C.); fazio@unime.it (E.F.); giacoma.brancato@studenti.unime.it (G.B.); deborah.lafauci@unime.it (D.L.F.);; 2 Department of Animal Medicine and Surgery, Faculty of Veterinary, Cardenal Herrera-CEU University, Alfara del Patriarca, 46115 Valencia, Spain; ksatue@uchceu.es

**Keywords:** Standardbred, ACTH, cortisol, training, racing

## Abstract

The hypothalamic-pituitary-adrenal (HPA) axis is a neuroendocrine system involved in the coping response to stressful challenges during exercise stimuli. Exercise represents a significant disruptor of homeostasis, inducing an ACTH-cortisol co-secretion, based on different characteristics of exercise in sport horses. Based on this statement, the aim of this study is to evaluate the circulating adrenocorticotropin and cortisol changes in Standardbred trotters, after training and racing sessions, considering the different age and sex. In particular, the aim is to determine to what extent the level of ACTH and cortisol increases during maximum effort in competition conditions (racing), and to compare two exercise conditions of different intensity, training and racing sessions, and effects on ACTH and cortisol responses. Ten Standardbreds, three females and seven males, clinically healthy, were enrolled and subjected to two exercise conditions: a non-competitive session (training) and then a competitive event (racing). Four of them were 2-year-olds and a further six were 3-year-olds. Training and racing effects on both ACTH (*p* < 0.01) and cortisol (*p* < 0.01) values were obtained. Compared to the training session, horses showed greater ACTH concentrations at rest (*p* < 0.001), at 5 (*p* < 0.01) and 30 min (*p* < 0.001), and lower cortisol concentrations only at rest (*p* < 0.01) after racing; 2- and 3-year-old horses showed the greater ACTH concentrations at 5 and 30 min (*p* < 0.01) post-racing; males showed the greater ACTH concentrations at 5 min and 30 min (*p* < 0.01) post-racing. The different stimuli of the two contexts, and differences in exercise intensity, such as training and competitive event, may have affected the direction of hypothalamic-pituitary-adrenal (HPA) axis response, both as an ability to adapt to physical stress of different intensity and as a preparatory activity for coping with stimuli. In conclusion, training and racing events induced a different HPA axis response in which both emotional experience and physical maturity could induce a significant adaptive response. As ACTH and cortisol concentrations in adult equids are extremely heterogeneous, further investigation is required to explore how different variables can influence the hormonal dynamics and their role as expressions of adaptive strategies to stress in horses.

## Simple Summary

This is an experimental study carried out on 10 trained Standardbreds, aged two and three years, including three females and seven males, with two main objectives: firstly, to examine the adrenocorticotropin (ACTH) and cortisol responses to training and racing sessions at rest condition, and at 5 min and 30 min after the training and racing sessions; secondly, to evaluate the effect of age and sex on endocrine parameters in both sessions. The effect of training and racing on ACTH (*p* < 0.01) and cortisol (*p* < 0.01) concentrations was obtained. Compared to the training session, horses showed greater ACTH concentrations at rest (*p* < 0.001), at 5 (*p* < 0.01) and 30 min (*p* < 0.001), and lower cortisol concentrations only at rest (*p* < 0.01) after racing; 2- and 3-year-old horses showed the greater ACTH concentrations at 5 and 30 min (*p* < 0.01) post-racing; males showed the greater ACTH concentrations at 5 min and 30 min (*p* < 0.01) post-racing.

## 1. Introduction

Exercise is a stressor that involves many regulatory endocrine systems that prompt the body to adapt and achieve a new dynamic equilibrium to maintain body homeostasis [1]. These adaptive endocrine responses are mainly expressed through the activation of the hypothalamic-pituitary-adrenal (HPA) axis contributing to include mechanical, metabolic, cardiovascular, and behavior modifications [2]. Several reports, conducted on sport horses and other athletic species in different exercise conditions, have highlighted the physiological and behavioral mechanisms by which HPA axis function influences stress adaptation. Primarily cortisol, among the HPA axis’s hormones, increases hepatic gluconeogenesis and promotes lipolysis to provide fuel for prolonged, and submaximal exercise. Hence, ACTH and cortisol are both expressions of anabolic and catabolic balance and their concentrations often increase in response to stress and/or exhaustion; in fact, these conditions cause an increase in both circulating hormones [3]. Physical exercise represents a significant and powerful disruptor of homeostasis, inducing a consensual ACTH-cortisol co-secretion, based on the influence of type, intensity and duration of physical activity in sport horses [4,5,6,7], dogs and humans [8,9,10]. The individual fitness, the training degree [11], and age [12], and the novelty stimulus surge of the ACTH sets [13], along with the degree of previous experience in competitive racing [14] could affect the magnitude of ACTH and cortisol profiles. Moreover, the evaluation of HPA axis hormones has been used to assess training status, performance degree [15,16,17,18,19,20], and overtraining syndrome of athletic horses [12,18,19,20,21,22,23,24,25,26,27,28], sport humans [9,29], and, specifically, Standardbreds’ performance [30,31,32]. Several reports have highlighted that long-term exercise increases cortisol concentrations, but short-term low-intensity exercise has not been shown in cortisol changes, or only slightly [33,34,35]. Interestingly, in the last decade the attention of scholars has been focused on the evaluation of salivary cortisol concentrations during different types of exercise and race training sessions [36,37,38]. Several reports have been published on how to reduce stress, and on the measurement of cortisol concentration through the non-invasive method of saliva [36,37,39,40,41,42]. Specifically, Strzleec et al. [36] reported that cortisol concentration decreases after dressage and that exercise intensity does not affect horses’ cortisol concentrations, concluding that stress can be reduced by moderate intensity and duration of exercise. Therefore, differences in exercise intensity, such as those between walk, trot and light canter, do not influence the horse’s stress. However, more research is needed to better understand the mechanisms of the adaptive responses of different types of sport horses during their athletic performance. On this basis, it has been hypothesized that different hormonal responses to non-competitive training and competitive sessions could occur, and that differences in exercise intensity may have affected the direction of hypothalamic-pituitary-adrenal (HPA) axis response, according to age and sex variables. Hence, the aim of this study was to determine to what extent the level of ACTH and cortisol in 2- and 3-year-old male and female Standardbreds increases during a race and to compare circulating ACTH and cortisol profiles after training and racing sessions.

## 2. Materials and Methods

### 2.1. Animals

All methods and procedures used in this study followed the guidelines of Italian law (D.L. 04/3/2014 n. 26) and the EU directive (2010/63/EU) on the protection of animals used for scientific purposes and approved by the Animal Ethics Committee for the Care and Use of Animals of University of Messina, Italy. (No. ME08/2023).

Two exercise conditions of different intensity (training and racing) were performed on 10 enrolled Standardbred racehorses: three females and seven males. Four of them were 2-years old and a further six were 3-years old and weighed (mean ± standard deviation) 384 ± 42 (range 331–485) Kg. All horses were kept in individual stalls bedded on wood shavings and allowed to move freely in a sand paddock 5 h/day. The horses were offered a diet consisting of grass (~8–10 kg/horse) and a commercial pelleted grain (~2 kg/horse) was fed and split into two feeds offered at 08.00 and 14.30 h. Horses were fed with diet formulated to meet the 1989 NRC for requirements horses. The concentrate portion of the ration contained 3.08 Mcal/kg digestible energy and 18% crude protein, while the hay contained 2 Mcal/kg digestible energy and 7% crude protein. Height at withers and total body weight were measured. For each horse, a body condition score (BCS) was evaluated on a scale of 1 to 9, with 1 being extremely emaciated and 9 extremely fat as reported by Henneke et al. [43]. Horses maintained body mass within 2–3% for a minimum of two weeks prior to the start of the study and all had body condition scores of 5–7 [43]. Horses remained healthy throughout the study with no change in BCS. Salt blocks and water were available ad libitum.

The general health state of the horses, based on thorough clinical and orthopedic examination, was assessed prior to the exercise 1 and 2. Physical examinations did not reveal clinical signs of any disease. All horses were in regular training. Although the participating horse and rider teams had a range of previous experience, all had competed in national rides in the past. This study was carried out during the spring, at the racecourse “La Favorita”, located in Palermo (Sicily, Italy): 38°07′55″ N Longitude: 13°20′08″, elevation above sea level: 46 m, which has a 1000 m long oval racetrack. During the experiments, horses were exposed to similar environmental conditions; the external conditions were optimal with a dry and firm track, sunny but cool weather and no wind. Ambient conditions measured from times 14.00 h to 16.30 h at the racecourse were moderate throughout the course of both exercises. Air temperature ranged from 17 to 21.5 °C (the highest at being at 14.30 h); relative humidity ranged 40.5–55.5 (the highest being at 15.00 h). The weather conditions on the study days were similar, with a temperature of between 18 and 20 °C, moderate windiness (4–6 m/s) and relative humidity between 40 and 60%. Other data collected from the “ride cards” included heart rate (HR), respiratory rate (RR) and rectal temperature (RT).

Heart rate was measured using a pulsimeter (Polar S710i™, Polar Electro Oy, Kempele, Finland) continuously during both exercises. The HR at rest, at end of exercises and 15 and 30 min of recovery were recorded using a phonendoscope. Respiratory rate was recorded at rest, after the 500 m finish (within 2 min after finishing the exercise), and at 15 and 30 min post exercise.

The RT was measured by a commercial digital thermometer (Microlife Ag, Widnau, Switzerland) before both exercises at rest, at end of exercises, and at 15 and 30 min of recovery.

Horses were submitted to two different exercise conditions: a non-competitive training race (exercise 1) and then, after 3 days, a competitive event (exercise 2), according to similar protocols suitable for their performance activity. Both exercise sessions were performed between 14.00 and 17.30 h. The same drivers sat on a sulky towed by the horse both during training and racing sessions (Table 1).

During the training session, the horses performed two racetrack rounds of “strenuous training” at the speed of 8–10 m/s for 5 min. Then, they took part in a “sprint training”, during which they ran 1600 m, reaching a speed of 10–12 m/s. At the end, the horses carried out two “basic training” rounds at a speed of 5–8 m/s. For each phase of training, the driver kept track of speed to keep it constant using the distance marked by the stakes on the sidelines. At the end of the physical activity, each horse was brought to the stable, where the harness was removed. At this stage, each horse was submitted to a 10 min walking cool-down phase. Therefore, the full training lasted 30 min, including the cool-down phase.

The racing session was performed over 1600 m. Before the race, each horse performed a 5 min warm-up at the speed of 8–10 m/s, making two racetrack rounds of sprint training. During the race, the driver pushed the horse to the maximal exercise level, reaching the highest average speed of 15–17 m/s at the initial and the arrival phases. Subsequently, a basic training round at the speed of 5–8 m/s was carried out. At this stage, the horse was submitted to a 10 min walking cool-down phase. Therefore, the physical activity during the race lasted 30 min, including the cool-down phase.

### 2.2. Blood Sampling and Hormone Analyses

Blood samples (10 mL) were collected from the jugular prior to both exercise sessions, in basal condition, at 2 p.m. and 5 min after the training and race sessions, before the cool-down phase at 5 p.m. At the end, samples were collected 30 min after the exercise, including the cool-down phase, at 5.30 p.m. On the day of blood sampling, no restraint was necessary as the horses were already familiar with handling procedures. Informed consent from horse owners was provided.

Plasma ACTH was measured in unextracted plasma samples, and the concentrations were analyzed in duplicate using a commercially available radioimmunoassay kit (ELSA-ACTH, CIS-BioInternational, Gif-sur-Yvette, France) that have been validated for use on equine samples [44]. The hormone assay used has a range for ACTH detected of 0–440 pmol/L. The sensitivity of the assay ACTH was 0.44 pmol/L. The intra-assay and interassay coefficients of variation were 6.0% and 15.0%, respectively.

To analyze cortisol concentrations, blood samples were centrifuged at 3000× *g* for 15 min and the obtained serum samples stored at −20 °C until analyzed. Total serum cortisol concentrations were analyzed in duplicate using a commercial competitive enzyme assay (Enzyme Immunoassays, Roche Diagnostics GmbH, Mannheim, Germany) and an automated analyzer (BRIO, SEAC, Rome, Italy). During the first incubation, the cortisol sample competed with cortisol conjugated to horse radish peroxidase for the specific sites of the antiserum coated on the wells. Following incubation, all unbound material was removed by aspiration and washing. The enzyme activity bound to the solid phase is inversely proportional to cortisol concentration in calibrators and samples and is made evident by incubating the wells with a chromogen solution (tetramethylbenzidine) in substrate-buffer. Colorimetric reading was carried out using a spectrophotometer at 450, 405 nm wavelength (Sirio S, SEAC, Florence, Italy). The assay sensitivity was 5 ng/mL. The intra- and interassay CVs were 4% and 6.9%, respectively. All assays were performed according to the manufacturer’s instructions. All samples were immediately processed in the adjacent laboratory at the racecourse and then harvested and stored in polystyrene tubes at −20 °C until analysis within one week.

### 2.3. Statistical Analysis

Data were presented as mean ± standard deviation (S.D.) of duplicate measurements in tables with the respective international units (UI) for the parameters studied. Normality was verified in all the data, using the Kolmogorov Smirnov test. In order to account for the different physiological variables, statistical analysis was performed by one-way analysis of variance (ANOVA) to evaluate age, sex and exercise effects on hormonal changes over basal time points in all sport horses.

A two-way repeated measures analysis of variance (Two-way RM ANOVA) was applied on the physiological changes, in order to test the effects of training and racing sessions together with their possible interaction. Post-hoc comparisons were performed using Tukey’s test. The level of significance was set at *p* < 0.05. All calculations were performed using the PRISM package versions 10.3.0 (GraphPad Software Inc., San Diego, CA, USA). Also, percentage differences (Δ%) between after vs. at rest values and between racing session vs. training session values were also calculated.

## 3. Results

**Figure 1:**
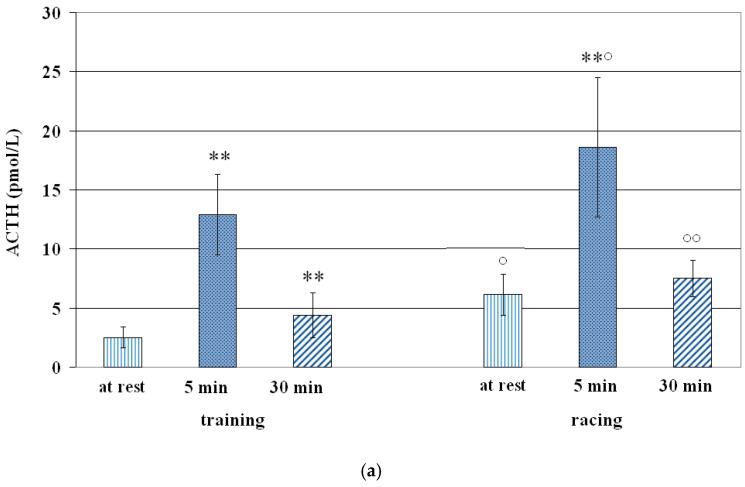

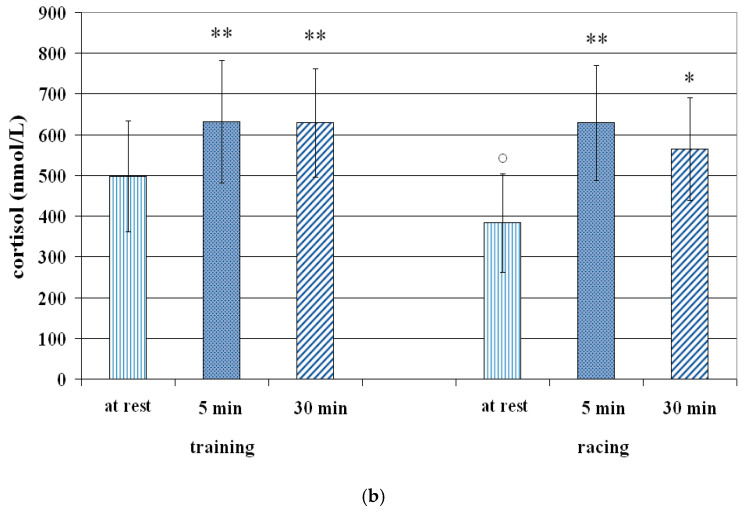
Circulating ACTH (**a**) and cortisol (**b**) concentrations (Mean ± S.D.) of Standardbreds before and after training and racing sessions. Asterisk indicates significant differences vs. at rest values * *p* < 0.01; ** *p* < 0.001. Symbol indicates significant differences vs. training ° *p* < 0.01; °° *p* < 0.001.

**Figure 2:**
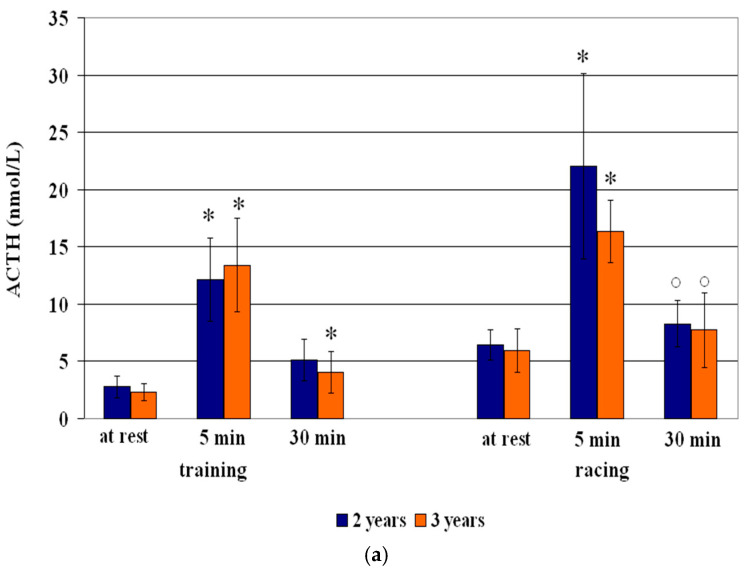

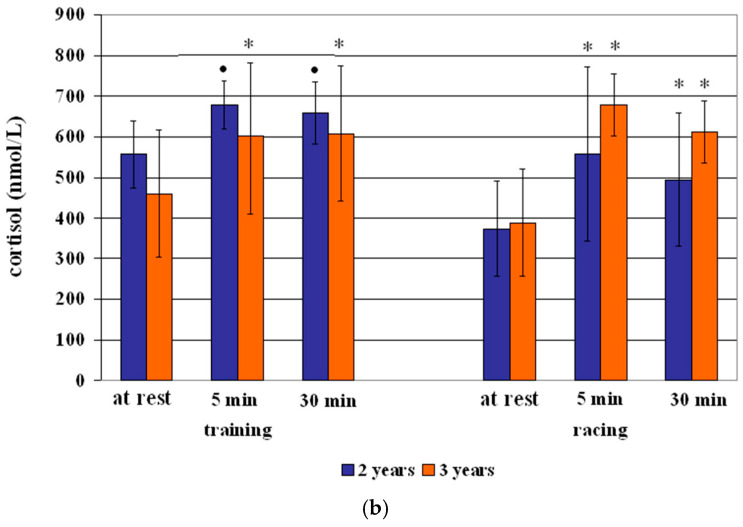
Circulating ACTH (**a**) and cortisol (**b**) concentrations (Mean ± S.D.) of Standardbred 2- and 3-year olds before and after training and racing sessions. Asterisk indicates significant differences vs. at rest values * *p* < 0.01. Symbol indicates significant differences vs. training ° *p* < 0.01. Symbol indicates significant differences vs. 3-years-old • *p* < 0.01.

**Figure 3:**
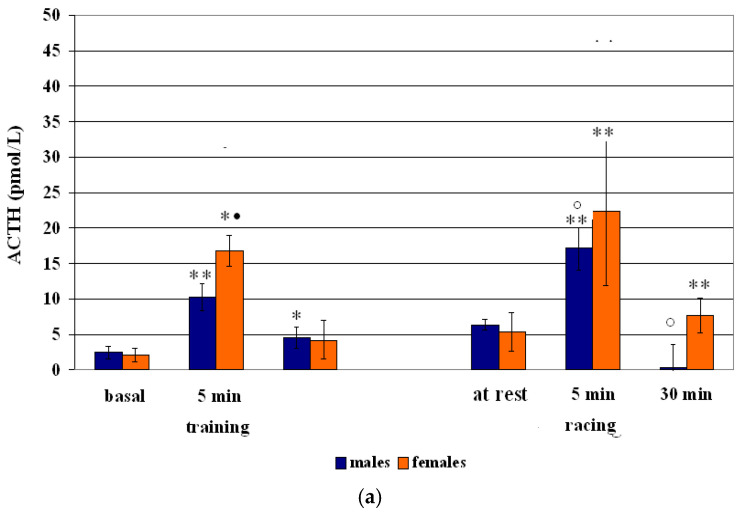

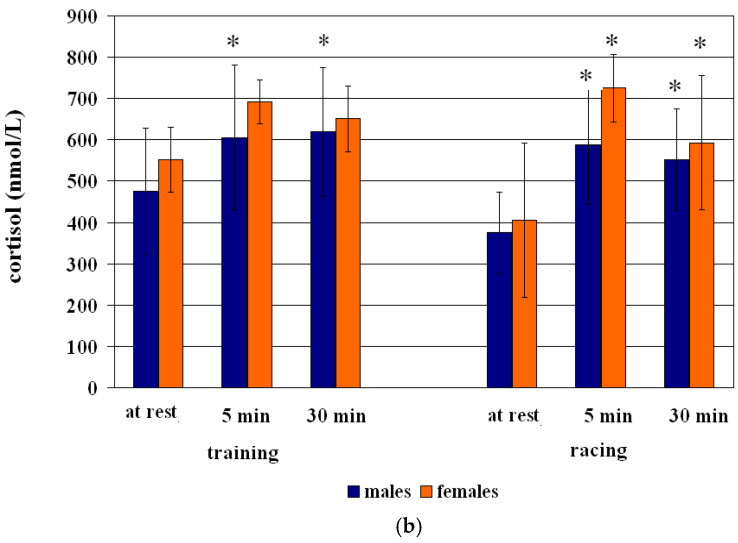
Circulating ACTH (**a**) and cortisol (**b**) concentrations (Mean ± S.D.) of male and female Standardbreds before and after training and racing sessions. Asterisk indicates significant differences vs. at rest values * *p* < 0.01, ** *p* < 0.001. Symbol indicates significant differences vs. racing 1 ° *p* < 0.01 Symbol indicates significant differences vs. males • *p* < 0.01.

Means (mean ± S.D.) of ACTH and cortisol concentrations are presented in Table 2, Table 3 and Table 4 and in Figure 1, Figure 2 and Figure 3.

### 3.1. ACTH-Cortisol Training Session Effect

The comparison of results obtained in this study with published data reported for horses in the literature did not reveal any large discrepancies and were in agreement with those physiological wide ranges; slight differences could be ascribed to differences in laboratory analysis techniques. However, data obtained confirm the trends recorded both in training and racing conditions [4,5,6,26,27,28,45,46,47].

Compared to resting values, a significant increase of ACTH concentrations 5 min (+414%; *p* < 0.001) and 30 min +76%; *p* < 0.001) after training, and of cortisol concentrations 5 min (+27%; *p* < 0.001) and 30 min (+66%; *p* < 0.001) after training was observed (Table 2 and Figure 1). A significant effect of training sessions both on the ACTH (F = 55.70; *p* < 0.01) and cortisol (F = 27.70; *p* < 0.01) changes was recorded.

Regarding the age effect, 2-year-old Standardbreds showed the greater ACTH concentrations at 5 min (+336%; *p* < 0.01) and at 30 min (+83%; *p* < 0.01), and, likewise, 3-year-olds showed greater concentrations at 5 min (+480%; *p* < 0.01) and 30 min (+75%; *p* < 0.01), compared to resting values. Significant effects of training on the ACTH concentrations both in 2-year-old horses (F = 28.73; *p* < 0.01) and 3-year-old ones (F = 29.80; *p* < 0.01) were obtained (Table 2 and Figure 1).

Three-year-old horses showed greater cortisol concentrations both at 5 min (+31%; *p* < 0.01) and 30 min (+32%; *p* < 0.01), compared to resting values. A significant effect of training on the cortisol concentrations only in 3-year-old horses (F = 39.57; *p* < 0.01) was obtained. Compared to 3-year-old horses, 2-year-old ones showed greater cortisol values (*p* < 0.01) at 5 min and 30 min.

Related to the sex effect, males showed greater ACTH concentrations at 5 min (+314%; *p* < 0.001) and 30 min (+82%; *p* < 0.01) after training, and females at 5 min (+697%; *p* < 0.01) and 30 min (+98%; *p* < 0.01) then resting values (Table 2 and Figure 1). A significant effect of training on the ACTH changes both in males (F = 47.30; *p* < 0.01) and in females (F = 10.66; *p* < 0.01) was obtained. The comparison between males and females showed the greatest ACTH concentrations at 5 min (*p* < 0.01) in females.

Related to cortisol concentrations, males showed greater concentrations both at 5 min (+27%; *p* < 0.01) and 30 min (+30%; *p* < 0.01), compared to resting values, with a significant effect of training on the cortisol of males (F = 19.02; *p* < 0.01) (Table 2 and Figure 1).

### 3.2. ACTH-Cortisol Racing Session Effect

Compared to resting values, an increase of ACTH concentrations at 5 min (+203%; *p* < 0.001) and 30 min (+23%; *p* < 0.01) after racing, and of cortisol at 5 min (+64%; *p* < 0.001) and 30 min (+47%; *p* < 0.01) after racing was observed (Table 3 and Figure 2). A significant effect on the ACTH (F = 42.17; *p* < 0.01) and cortisol (F = 16.45; *p* < 0.01) changes was recorded.

Regarding the age effect, both 2-year-old (+243%; *p* < 0.01) and older (+176%; *p* < 0.001) Standardbreds showed greater ACTH concentrations at 5 min (*p* < 0.01), compared to the resting values (Table 3 and Figure 2). A significant effect of racing both in 2-year-old horses (F = 21.47; *p* < 0.01) and in the older horses (F = 31.41; *p* < 0.01) was observed

Both 2-year-old and 3-year-old horses showed increases of cortisol concentrations at 5 min (+48% and +74%, respectively; *p* < 0.01) and 30 min (+32% and +57%, respectively; *p* < 0.01), compared to resting values (Table 3 and Figure 2). A significant racing effect on cortisol changes in younger (F = 14.35; *p* < 0.01) and older horses (F = 7.75; *p* < 0.01) was obtained.

Regarding the sex effect, males showed greater ACTH concentrations at 5 min (+172%; *p* < 0.001), and females both at 5 min (+68%; *p* < 0.001), and 30 min (+44%; *p* < 0.001), than resting values (Table 3 and Figure 2), with a significant effect of racing on the ACTH changes in males (F = 49.95; *p* < 0.01) and females (F = 10.66; *p* < 0.01).

Compared to resting values, males showed an increase in cortisol concentrations at 5 min +56%; (*p* < 0.01) and at 30 min (+47%; *p* < 0.01), and females showed an increase of cortisol concentrations at 5 min (+79%; *p* < 0.01) and at 30 min (+46%; *p* < 0.01) (Table 3 and Figure 2). A racing effect was observed on the cortisol changes in males (F = 9.24; *p* < 0.001) and females (F = 27.87; *p* < 0.01).

### 3.3. Racing Session vs. Training Session

Compared to the training session, horses showed greater ACTH concentrations at rest (*p* < 0.001), at 5 (*p* < 0.01) and 30 min (*p* < 0.001), and lower cortisol concentrations only at rest (*p* < 0.01) after racing; 2- and 3-year-old horses showed the greater ACTH concentrations at 5 and 30 min (*p* < 0.01) post-racing; males showed the greater ACTH concentrations at 5 min and 30 min (*p* < 0.01) post-racing (Table 3 and Figure 2).

### 3.4. Functional Variables

Heart rate (HR) and respiratory rate (RR) increased at the end of training and racing sessions, remaining greater at 15 min and 30 min compared to resting values (*p* < 0.05). Related to rectal temperature (RT), increases at the end and at 15 min were recorded returning to a t rest values at 30 min of recovery (*p <* 0.05) (Table 3).

## 4. Discussion

This research shows that different endocrine responses to non-competitive and competitive exercise conditions happen according to different ages and sex. ACTH and cortisol increase after the training and racing sessions, confirming previous data recorded in athletic horses after physical exercise, in which both hormones rise between 5 and 30 min after the end of exercise in Thoroughbred horses [23,48], Warmblood horses [16], and showjumping horses [4,25].

ACTH and cortisol concentrations, detected at rest time, were significantly greater, by +145% and +38%, respectively, prior to the racing than prior to the training session; this could be due to the psychological effect of waiting before the race start, as the role of affective processes underpinning temperament, mood and emotional reaction in determining discipline-specific performance [49].

The present study showed that ACTH concentrations at 5 min and 30 min post-racing exercise were 44% and 70% greater than at the same time post-training exercise. The same but more contained trend was recorded for cortisol concentrations at 5 min and 30 min post-exercise 2 that were 7% and 19% greater than at the same time post-training exercise.

A similar result was recorded by Nagata et al. [23], in which the greatest ACTH concentrations were observed at 5 and 30 min after the incremental treadmill exercise in Thoroughbred horses. In this study [23], plasma ACTH responses during exercise were more sensitive to the intensity of exercise than its duration.

Our results are in line with those reported in a recent study that showed an anticipatory response before racing in Standardbred racehorses, as shown by significant differences in pre-training (97.3 ± 16.4 nmol/L) and pre-race cortisol concentrations (171.8 ± 18.7 nmol/L), respectively [50]. Furthermore, other authors have shown that the participation of horses in equestrian competitions leads to an activation of the hypothalamus-adrenocortical function, reflected by a transient increase in cortisol release, and an increase in sympathoadrenal activity, indicated by a rise in heart rate [26].

In our experimental conditions, the training session speed was about 10–12 m/s and the racing session speed was about 15–17 m/s. Kurosawa et al. [22] reported that the onset time and the size of ACTH rise as a response to the SET on treadmill were significantly correlated to the speed sustained by Thoroughbred horses. Based on ACTH increase, Kurosawa et al. [22] stated the ACTH should be considered as a marker of stress and performance during physical exercise in Thoroughbred horses.

Our results are in agreement with Kurosawa et al. [22], since the HPA axis response of Standardbred was modulated by the increasing exercise intensity. In fact, our data showed a consistent effect of racing on the ACTH concentrations prior to the start, at 5 and 30 min after the race, compared to those of training. Moreover, previous studies found that not only ACTH response was more sensitive to the intensity of exercise, but it also showed a significant correlation with blood lactate concentrations [23] and with circulating arginine vasopressin concentrations during incremental exercise tests [13] Hada, 2003. The increase of ACTH values after 5 and 30 min of racing is also in agreement with ACTH increase recorded in Thoroughbreds submitted to the standardized exercise test (SET) on treadmill [16] Marc, 2000. However, submaximal treadmill exercises produced unmodified ACTH concentrations in young horses [20] McCarthy, 1991.

In young Friesian horses, a significant difference in the net increase of circulating ACTH concentrations was recorded after dressage training sessions; nevertheless, no difference in the post training values of cortisol concentrations was found [28] Fazio, 2016. Physical training clearly stimulated ACTH secretion, but, as previously recorded in a study by Ferlazzo et al. [25], an increase in circulating cortisol concentrations did not directly reflect differences in ACTH secretion, indicating that there was a differentiated response of the two hormones under the same conditions, probably related to different responses to mental exercise-related stress. The magnitude of ACTH changes after the first training session suggested that the novelty of stimuli represented an early stress manifestation in horses, thus inducing an initial, marked arousal response to stress, especially at the training start day; what is more, this result confirmed previous studies which showed that new stimuli, during exercise tests, increased plasma ACTH in Thoroughbred horses [13].

The participation of horses in competitive or non-competitive races caused an activation of HPA functions, reflected in a transient increase in cortisol release. Cortisol secretion is generally caused by an endogenous release following ACTH stimulation. This response during riding is at least in part due to the physical activity required of the horse. In agreement with previous studies, cortisol concentrations showed a significant increase after training and racing sessions. It is recognized that both a moderate exercise, like the training session of the present study, and an intense exercise, like the racing session, stimulate the cortisol releasing [50].

In fact, some authors showed a cortisol increase in response to an incremental exertion [19], after different types of sporting event competitions, including cross-country [50], endurance training sessions, competitions for Arabian horses [51], and after SET on the track [52]. Other authors have shown a 100% and 200% post-exercise increase in cortisol concentrations compared to rest values in horses submitted to endurance races [25] Ferlazzo, 2012. The increase of cortisol values at 5 and 30 min after racing is in agreement with a previously recorded cortisol rise after physical activity in trotters [53] Lindner 2002. Despite the different types of exercise, however, 5 min post-race the trend of cortisol, compared to that of ACTH, showed a rise comparable to that reported in post-training session. This result was also recorded by other authors that showed an increase of cortisol concentrations after dressage in both horses and riders [54].

The best recovery time was recorded during the training session; in fact, cortisol concentrations at 30 min post-racing exercise persisted at the high levels, quite comparable to those recorded at 5 min. This could be due to a major emotional impact and physical effort, characterizing a competitive race session. Previously, an increase of cortisol concentrations at 40–60 min post-race was reported in trotters [55]. Other authors showed that in Standardbred horses the cortisol significantly takes a cut 4–8 h after the exercise and then it needs about 24h to return at basal values [11]. Jumping horses showed a slower recovery of cortisol in the group of animals jumping the higher fences, with higher cortisol concentrations at 30 min post exercise than those of 5 min [25]. In addition, a significant cortisol increase before and after show jumping events in sport horses previously transported was recorded; moreover, the cortisol increases after the competitive event showed a correlation with the exercise rather than the transport stress [6]. Rivera et al. [56], reported high cortisol values at 15 min post-endurance training, with a decrease after 75 min.

Based on our results, the effect of circadian rhythms on ACTH and cortisol trends can be excluded; in fact, the blood samples were collected at the same times of the afternoon (from 3.00 to 5.30 p.m.). It is well known that exercise influences the regular circadian rhythms of cortisol secretion, which in the horse is greater in the morning than in the evening, with an acrophase at 10:50 a.m. [57]. A similar position is accepted in humans as well, since the cortisol response to exercise is significantly modulated by time of day; consequently, neglecting the circadian cortisol kinetic may introduce errors into conclusions about the hormonal response to exercise [58]. Regarding the age effect on ACTH and cortisol changes, the lower concentrations of both hormones in 2-year-olds than older horses at 5 min after the training session, and for cortisol at 5 and 30 min after, are in agreement with previous studies carried out on Standardbreds after SET on the oval dirt track [52]. Interestingly, albeit not significant, higher cortisol concentrations at all the times post-race compared to post-training in the younger horses were also recorded. One explanation could be due to the superimposed effort of both training and race sessions from each other. It is possible that the younger subjects spent a greater amount of energy than the older ones, which probably had better restorative ability and exercise adaptation capacities, as result of long-time training. A recent study showed that in Arabian racing horses, cortisol concentration tended to gradually increase after the subsequent training sessions during the racing season, and the best performances were achieved by the animals with lower cortisol concentrations [7]. After all, in human runners, the adaptation capacity of HPA axis was also demonstrated; cortisol concentrations were lower in trained than in untrained subjects under incremental exercise. Furthermore, the untrained controls showed an elevated cortisol concentration at the end of each intensity level; hence, trained athletes only had a significant increase in cortisol concentration after an exhaustive exercise session [20].

Age and training state positively influenced the locomotion parameters in Thoroughbreds and related metabolic costs related to competitive physical activity [59]. In addition, based on available data in the literature, Thoroughbred racehorses showed higher cortisol values in 2-year-olds than in the older horses [17]. It is possible to presume that the greater responsiveness of the HPA axis in the younger subjects was correlated to less experience in competitive race sessions, according to the greater emotional impact due to the novelty stress represented by the competitive event [13].

Although all horses in this work were adult, it is well documented that the age, genetic and environmental factors can impair the reactivity capacity of the HPA axis. The aging can also affect the hormonal responses usually put in place to cope with physical exercise, changing the control ability of the body on the cardiovascular function, the metabolism and substrate utilization [60]. Indeed, the authors showed that in Standardbred mares the HPA axis experience is submitted to a functional decline with age although the exact mechanisms remain unknown, while exercise training can facilitate the counteraction of these progressive deficits [30].

Regarding the influence of sex, the lack of significant differences in ACTH concentrations between males and females, during both training and racing, was reported. The cortisol concentrations showed the lower values in males compared to females, at all times and after both the training and the race sessions, but without a significant difference. The reason for this sex difference in the axis modulation in response to physical effort is not clear, so more investigations are needed.

### Limitations of the Current Investigation

The clear limitation of this investigation is the small group of horses that completed the study, unfortunately related to events that occurred during the experiment. An additional 10 potential horses that were due to participate were excluded, due to retirement from racing, as were 10 because trainers did not want the horse to complete the exercises required by the study. Another limit is the lack of use of a heart rate monitor during both exercises to estimate speed and evaluate trot parameters, acceleration and distance covered, necessary for the assessment of fitness and performance. Furthermore, another limitation could be plasma cortisol sampling versus the non-invasive salivary cortisol method and the lack of some stress parameters (lactate, PCV, haematocrit). Unfortunately, this was a choice linked to the lack of authorization from race authorities and several owners and riders: it being a national competition forced us to reduce the analysis on the horses by allowing us only a single plasma sample from which it was possible to determine the measurement of both plasma parameters studied.

## 5. Conclusions

Different exercise intensities, in the two contexts of training and competition, induced the direction of the HPA axis response. This may influence the ability of horses to adapt to physical stress. It is reported in the literature that many factors can influence ACTH and cortisol levels. Measurement of basal ACTH levels is influenced by several pre-analytical factors and, in the context of low intensity exercise, significant increases have been reported after only 30 min of exercise. In order to optimize physical fitness in horses, further research is needed to explore how different variables (e.g., emotional experience, age, sex, sample management, etc.) may influence the ability to adopt stress coping strategies in performance horses.

## Figures and Tables

**Table 1:** Scheme of training and racing protocols.

Training	Racing
Two rounds: strenuous training: velocity 8–10 m/s; duration 5 min	Two rounds: strenuous training: velocity 8–10 m/s; duration 5 min
1600 m: sprint training: velocity 10–12 m/s; duration 2.40 min	1600 m: sprint training: velocity 15–17 m/s; duration 1.58 min
Two rounds: basic training: velocity 5–8 m/s; duration 10 min	One round: basic training: velocity 5–8 m/s; duration 10 min
Cool down at the pass: duration 10 min	Cool down at the pass: duration 10 min

**Table 2:** Plasma ACTH and serum cortisol concentrations of Standardbred horses before and after training session.

	ACTH (pmol/L)				
	at Rest	5 min	Δ%	30 min	Δ%
Total	2.50 ± 0.89	12.91 ± 3.43 ^b^	+416	4.40 ± 8.66 ^b^	+76
2-year-old	2.78 ± 0.95	12.13 ± 3.66 ^a^	+336	5.10 ± 1.80	+83
3-year-old	2.31 ± 0.78	13.40 ± 4.06 ^a^	+480	4.04 ± 1.83 ^a^	+75
males	2.48 ± 0.89	10.28 ± 1.89 ^b^	+314	4.51 ± 1.51 ^a^	+82
females	2.11 ± 0.94	16.81 ± 2.21 ^aB^	+697	4.19 ± 2.72	+98
	cortisol (nmol/L)				
Total	498 ± 136	632 ± 151 ^b^	+27	629 ± 133 ^b^	+26
2-year-old	555 ± 82	678 ± 59 ^A^	+22	659 ± 76 ^A^	+19
3-year-old	460 ± 158	601 ± 190 ^a^	+31	608 ± 165 ^a^	+32
males	475 ± 153	606 ± 175 ^a^	+27	619 ± 156 ^a^	+30
females	552 ± 79	692 ± 54	+25	651 ± 79	+18

Letters indicate differences vs. at rest: ^a^ (*p* < 0.01); ^b^ (*p* < 0.001); vs. 3-year-old ^A^ (*p* < 0.01); vs. males ^B^ (*p* < 0.01).

**Table 3:** Plasma ACTH and serum cortisol concentrations of Standardbred horses before and after racing session.

		ACTH (pmol/L)			
	at Rest	Δ%	5 min	Δ%	30 min
Total	6.13 ± 7.79 ^C^	+203	18.61 ± 26.82 ^Cb^	+23	7.52 ± 1.54 ^CD^
2-year-old	6.43 ± 6.13	+243	22.05 ± 8.10 ^Ca^	+29	8.29 ± 2.0 ^C^
3-year-old	5.92 ± 8.70	+176	16.32 ± 2.74 ^C^	+31	7.74 ± 3.28 ^C^
males	6.30 ± 3.52	+172	17.15 ± 3.05 ^bC^	+32	8.35 ± 3.25 ^C^
females	5.34 ± 12.35	+68	22.29 ± 10.41 ^b^	+44	7.67 ± 2.41 ^b^
	cortisol (nmol/L)				
Total	383 ± 121 ^C^	+64	629 ± 141 ^b^	+47	565 ± 126 ^a^
2-year-old	375 ± 118	+48	57 ± 215 ^a^	+32	494 ± 165 ^a^
3-year-old	389 ± 132	+74	678 ± 77	+57	612 ± 77
males	375 ± 99	+56	587 ± 143 ^a^	+47	552 ± 123 ^a^
females	405 ± 187	+79	725 ± 82 ^a^	+46	593 ± 162 ^a^

Letters indicate differences vs. at rest: ^a^ (*p* < 0.01); ^b^ (*p* < 0.001); vs. training ^C^ (*p* < 0.01); ^D^ (*p* < 0.001).

**Table 4:** Mean ± S.D. for heart rate (HR), respiratory rate (RR) and rectal temperature (RT) at rest, at the end of training and racing sessions, and at 15 min and 30 min of recovery.

		Training		
	At Rest	End	15 min	30 min
HR (beats/min)	38 ± 10	180 ± 20 ^a^	80 ± 15 ^a^	65 ± 5 ^a^
RR (breaths/min)	18 ± 8	80 ± 5 ^a^	75 ± 10 ^a^	40 ± 10 ^a^
RT °C	37.5 ± 0.3	38.6 ± 0.3 ^a^	38.5 ± 0.2 ^a^	37.8 ± 0.4
racing				
HR (beats/min)	39 ± 11	189 ± 30 ^a^	84 ± 10 ^a^	55 ± 5 ^a^
RR (breaths/min)	18 ± 3	80 ± 5 ^a^	78 ± 10 ^a^	45 ± 8 ^a^
RT °C	37.6 ± 0.3	39.2 ± 0.4 ^a^	38.6 ± 0.3 ^a^	37.8 ± 0.4

^a^ Indicates a significant difference (*p* < 0.05) from resting values.

## Data Availability

The data that support this study will be shared upon reasonable request to be corresponding author.
